# Computational translation of drug effects from animal experiments to human ventricular myocytes

**DOI:** 10.1038/s41598-020-66910-0

**Published:** 2020-06-29

**Authors:** Aslak Tveito, Karoline Horgmo Jæger, Mary M. Maleckar, Wayne R. Giles, Samuel Wall

**Affiliations:** 1 Simula Research Laboratory, Fornebu, Norway; 2 Department of Physiology and Pharmacology, Faculty of Medicine, University of Calgary, Calgary, Canada

**Keywords:** Biophysics, Computational biology and bioinformatics

## Abstract

Using animal cells and tissues as precise measuring devices for developing new drugs presents a long-standing challenge for the pharmaceutical industry. Despite the very significant resources that continue to be dedicated to animal testing of new compounds, only qualitative results can be obtained. This often results in both false positives and false negatives. Here, we show how the effect of drugs applied to animal ventricular myocytes can be translated, quantitatively, to estimate a number of different effects of the same drug on human cardiomyocytes. We illustrate and validate our methodology by translating, from animal to human, the effect of dofetilide applied to dog cardiomyocytes, the effect of E-4031 applied to zebrafish cardiomyocytes, and, finally, the effect of sotalol applied to rabbit cardiomyocytes. In all cases, the accuracy of our quantitative estimates are demonstrated. Our computations reveal that, in principle, electrophysiological data from testing using animal ventricular myocytes, can give precise, quantitative estimates of the effect of new compounds on human cardiomyocytes.

## Introduction

Our main goal was to develop model-based methods evaluating whether measurements of drug effects on animal cardiomyocytes can be translated to corresponding drug effects on human cardiomyocytes. The analysis reveals how effects on membrane currents carried by the same ion channel protein in animal and humans can be translated in order to evaluate and understand human ventricular action potentials. This approach provides a framework revealing unwanted side effects early in the development of novel drug candidates. The usefulness of animal models in understanding human electrophysiology is unquestioned, but limited by the inherent difference between the human and animal action potentials; see e.g.^1–3^. Often, this gives rise to difficulties in direct translation; even if some of the ion channels involved are functionally nearly identical, the overall composition of membrane ion channels can differ substantially between species. Therefore, block of the same ion channel in two different species may yield very different perturbations of the respective action potentials.

This limitation has serious implications for the overall efficiency and effectiveness of drug development. Currently, this process takes 10 to 15 years, with an average cost of ~2.5 billion USD^4^. Preclinical development, including animal testing for safety predictions, accounts for ~60% of these costs^5^. However, despite this cost and effort, many promising candidate drugs exhibit toxicity that has not been predicted prior to clinical trials and, ultimately, emerging therapies^6^; clearly, more quantitatively predictive tools are needed. Assuming that the major currents underlying the ventricular action potential are governed by the same or similar ion channel alpha subunit in an animal and in a human heart cell, and that these proteins have comparable function and are relatively well understood^1,7–11^, our approach can be used to translate measured pharmacological effects on the action potential in animal models to those in healthy human adult cardiomyocytes.

Previously^12,13^, we have developed a theoretical approach which allowed translation of measurements obtained on human induced pluripotent stem cell-derived cardiomyocytes (hiPSC-CMs) to adult cardiomyocytes. In brief, we have demonstrated that after inverting hiPSC-CM data, and finding the effect of a drug candidate on every ion channel, these findings can be accurately mapped to the case of mature cardiomyocytes. Thus, the effect of the drug on adult human heart cells can be deduced from measurements of hiPSC-CMs. Our aim here is to show that a similar approach can be employed in translation from animal myocytes to human results.

In order to fully translate the effect of a drug from data obtained from either hiPSC-CMs or animals, the effect of the drug must be used to estimate parameters of the associated mathematical model. This is indeed a challenging process; see e.g.^14–16^ with significant uncertainty. Here, in order to focus on the trans-species mapping, we use the techniques developed in^13^, simplified considerably by assuming that we know which of the ion channels that are affected by a given drug. In this simplified case it is sufficient to only have data on the transmembrane potential.

## Results

Here, we demonstrate that it is possible to estimate the effect of a drug candidate on humans solely based on measurements of analogous effects on animal cardiomyocytes. The dog data presented in^7^ is employed together with our method to estimate the effect that dofetilide will have on humans. Also, in^7^, the effect of dofetilide on human cardiomyocytes was reported, allowing us to assess the quality of our estimates. Additionally, we used the data provided in^17^ on zebrafish to estimate the effect of the drug E-4031 on humans. While the effect on human cardiomycytes is not reported in^17^, this has been reported in^18,19^, which we again used to assess the accuracy of our estimates. Finally, we used rabbit data presented in^20^ to estimate the effect of sotalol on human myocytes; data on human cardiomyocytes in response to sotalol has been presented in^20,21^ and is used here for comparison and assessment.

### Quantitative translation from dog to human

**Figure 1:**
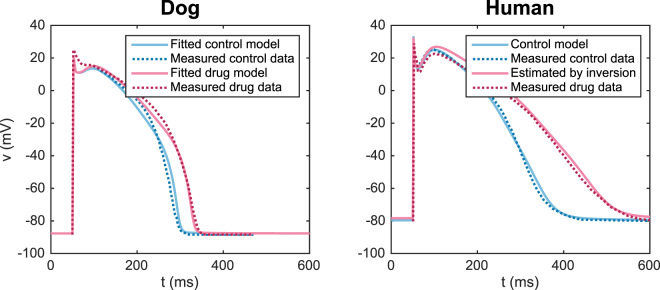
Dog (left) and human (right) ventricular action potentials in the control case and in the presence of 50 nM of the *I*_Kr_ blocker dofetilide. The dotted lines show measured data from^7^, and solid lines show simulation results. Note that the drug effect used in the simulation of the human AP was estimated from the dog data.

In Fig. 1, our method (see below) is applied to data published in^7^. In the left panel, experimental data (dotted) is shown for control (blue) and when 50 nM of the *I*_Kr_ blocker dofetilide is applied (red) to canine myocytes. Solid lines show the results of the mathematical model (Supplementary Information). The parameters defining a control model and an IC_50_ value representing the effect of the drug are estimated and used to model the no drug case (solid blue line) and when the drug has been applied (solid red line).

In the right panel, the control data (dotted blue) and the control mathematical model (solid blue) are illustrated for the human ventricular myocyte. We used the method to estimate the effect of the *I*_Kr_ blocker on the ventricular AP model of the dog, and this effect is translated (Supplementary Information) to the human AP model; this estimated AP (solid red) fits the measured data from^7^ very well.

In Table 1, we compare selected biomarkers (APD50 and APD90) for the control case of dog cardiomyocytes (measured), dog cardiomyocytes subjected to the drug (measured), the human control (measured), the human control (model), human cardiomyocytes subjected to the drug (measured) and human cardiomyocytes subjected to the drug (estimated as based on the dog data, as described in the Supplementary Information). All experimental data are taken from^7^. For the dog data, the APD50 is increased by 17.5% when the drug is added while, for human cells, the measured increase in APD50 is 33.3%. When we compare the output from the mathematical model for the control case, and the one where the effect of the drug is added to the model, we observe an increase of 38.0%. Thus, based only on data from the dog, our estimate of the increase of the APD50 for humans differs little from the measured value (38.0 vs 33.3%). The analysis of the APD90 also reveal a similarly small difference (40.2 vs. 44.3%). We conclude that the dog data on APD50/APD90 can be used to estimate the effect of the *I*_Kr_ blocker dofetilide on human cardiomyocytes well. Furthermore, the mathematical model estimates an IC_50_ value of 17 nM for block of *I*_Kr_ by dofetilide. This value is in good agreement with IC_50_ values for *I*_Kr_ block by dofetilide found in literature (1–70 nM^21–24^).

**Table 1:** APD values computed from measured and simulated action potentials in the control case and in the presence of 50 nM of the *I*_Kr_ blocker dofetilide.

	Dog, control measured	Dog, drug measured	Human, control measured	Human, drug measured	Human, control model	Human, drug estimated based on dog data
APD50 (ms)	201	+17.5%	230	+33.3%	221	+38.0%
APD90 (ms)	239	+18.3%	312	+44.3%	317	+40.2%

The cells in both the experiments^7^ and in the mathematical model were paced at 1 Hz.

### Quantitative translation from zebrafish to human

In Table 2, the same method was applied to data from zebrafish cardiomyocytes^17^. Again, we have focused on experimental APD50 and APD90 values for zebrafish in the control case and the increase caused by the application of 1 *μ*M of the *I*_Kr_ blocker E-4031. In addition, we report the increases in APD50/APD90 from measurements of human cardiomyocytes exposed to 1 *μ*M E-4031 from Bussek *et al*.^18^ (B) and Jost *et al*.^19^ (J).

**Table 2:** APD values computed from measured and simulated action potentials in the control case and in the presence of 1 *μ*M of the *I*_Kr_ blocker E-4031.

	Zebrafish control measured	Zebrafish drug measured	Human control measured	Human drug measured	Human control model	Human drug, estimated based on zebrafish data
APD50 (ms)	108	+38.7%	255 (B)	+27.7% (B)	221	+58.0%
				+54.2% (J)		
APD90 (ms)	144	+24.1%	356 (B)	+31.2% (B)	317	+62.8%
				+65.1% (J)		

The zebrafish data is from^17^ and the human data is from Bussek *et al*.^18^ (B) and Jost *et al*.^19^ (J).

Finally, we report the increases in APD50 and APD90 estimated for human cardiomyocytes as based on the measurements of zebrafish cardiomyocytes. Again, we observe that the APD changes estimated using the mathematical model for the human case agree well with the measured effects for human cells. For APD50, our method estimates a 58.0% increase, while the increase is 27.7% (B) or 54.2% (J) in the experimental data. Similarly, the method estimates a 62.8% increase of APD90, while the experimental data reports a 31.2% (B) or 65.1% (J) increase. Furthermore, the method estimates an IC_50_ value of 44 nM for block of *I*_Kr_ by E-4031, in line with corresponding IC_50_ values found in literature (10–397 nM^25–30^).

### Quantitative translation from rabbit to human

Table 3 shows the result when the same method is applied to rabbit ventricular myocyte data from^20^. In this case, two doses (10 *μ*M and 52 *μ*M) of the *I*_Kr_ and *I*_CaL_ blocker sotalol were employed. In addition^20,21^, provide measured data for human cardiomyocytes exposed to 30 *μ*M sotalol. The column at the extreme right in Table 3 reports the increases in human APD50 and APD90 estimated based on the rabbit data. Both the estimated increase in APD50 (24.7%) and APD90 (28.8%) seem to be in good agreement with the measured human values from^20,21^ (32.3% and 20.6% for APD50 and 37.5% and 28.2% for APD90). The method estimates an IC_50_ value of 12 *μ*M for block of *I*_Kr_ by sotalol and an IC_50_ value of 102 *μ*M for block of *I*_CaL_. The corresponding IC_50_ values found in literature are 51–343 *μ*M for *I*_Kr_^21–24^ and 193 *μ*M for *I*_CaL_^23^.

**Table 3:** Selected APD values from experimental and simulated action potentials in the control case and in the presence of the *I*_Kr_ blocker sotalol.

	Rabbit, control measured	Rabbit, drug measured	Human, control measured	Human, drug measured	Human, control model	Human, drug estimated based on rabbit data
APD50 (ms)	162	+21.2% (10 *μ*M)	185 (B)	+32.3% (30 *μ*M) (B)	221	+24.7% (30 *μ*M)
		+54.3% (52 *μ*M)	233 (O)	+20.6% (30 *μ*M) (O)		
APD90 (ms)	194	+19.6% (10 *μ*M)	235 (B)	+37.5% (30 *μ*M) (B)	317	+28.8% (30 *μ*M)
		+54.1% (52 *μ*M)	302 (O)	+28.2% (30 *μ*M) (O)		

The rabbit experimental data is taken from^20^, and includes the two drug doses 10 *μ*M and 52 *μ*M. The human experimental data is taken from Baczkó *et al*.^20^ (B) and Orvos *et al*.^21^ (O) and is measured for the drug dose of 30 *μ*M.

## Discussion

The use of animal models to aid in understanding of human anatomy and physiology is centuries old, see e.g.^31,32^.

In the case of the electrophysiology of the vertebrate heart, this type of comparative study^33^ has led to deep insight into key physiological mechanisms, but it has been difficult to use these models quantitatively due to inherent species differences. More specifically, while animal cells are routinely used as part of drug development, their quantitative usefulness is hampered by the significant differences in human and animal action potential waveforms. At present, the most useful results are obtained by utilizing observed correlations between human and animal biomarkers (see e.g.^34–38^). However, no mechanistic relationship has, to date, been derived to examine how drug candidates may affect human and animal cardiomyocytes differentially. This limits the predictive insights into the therapeutic or toxic effects on humans. In this study, we have shown how an antiarrhythmic drug effect on human ventricular myocytes can be revealed solely by measuring an analogous effect of the drug on animal ventricular myocytes. This new investigative capability opens a wide variety of possibilities to improve the testing of novel compounds.

Initially, we have shown that canine data can be used to reliably predict the effect of the drug dofetilide on human myocytes as judged by changes in the common biomarkers APD50 and APD90. Similarly, data from zebrafish action potentials was used to estimate the effect of E-4031 on human ventricular myocytes. Finally, the effect of sotalol on rabbit cardiomyocytes was used to estimate the effect of the same drug on human ventricular myocytes, and the results were revealing.

Our method can only be used to estimate a selected drug effect on ion currents that have significant impact on both the animal and the human action potentials. If one current is very small in an animal but very large in a human, drug effects on that current cannot be estimated using the present technique. One such case is illustrated in Fig. 1 of^1^, where the action potential of human, canine, rabbit, guinea pig and mouse are illustrated together with associated currents. The substantial differences between the human and mouse action potentials and underlying ion currents suggest that it is difficult to use our method to predict the effect of drugs on human cardiomyocytes by measuring the effect of the drug on mouse cardiomyocytes.

In the present study, our principal aim was to generalize the methods we have developed previously^12,13^ for extrapolating drug effects obtained using hiPSC-CMs to healthy, mature human ventricular myocytes. Importantly, we found that the an equivalent approach can be used to translate measured pharmacological effects on the action potential in animal models to those in healthy adult human myocytes by employing the same models for the same currents in different cell types. The assumption underlying this approach is that major currents are carried by the same or similar ion channels for which isoforms differences have been measured and are relatively well understood. This is indeed often the case. While sarcolemmal voltage gated ion channels are complex transmembrane proteins of several classes, often composed of more than one interacting or modifying subunit, the alpha subunit of cardiac sodium, potassium, and calcium channels do show very strong primary sequence similarities across species; genome comparisons highlight a number of highly conserved channel families including Nav, Kv, and Cav^39^. For instance, in epicardial adult ventricular myocytes, channels underpinning late repolarization (delayed rectifier currents rapid and slow, *I*_Kr_/hERG and *I*_Ks_/KvLQT-minK, respectively) are conserved among human, canine, guinea pig, and rabbit cells^8,40^. Again, the transient outward current, *I*_to_, prominent in early depolarization in the rabbit ventricle via Kv1.4/4.2/4.3, is similar in presentation and action potential effects in adult human myocytes, as carried by Kv4.3^9^. The inward rectifying K^+^ current (*I*_Kl_), the L-type Ca^2+^ current (I_CaL_), and the Na^+^/K^+^ pump current (I_NaK_) are also present and effectual in rabbit^10^ in addition to other species, as in human. Rabbit measurements of calcium handling and excitation-contraction coupling have long been used as a basis to study and model these important phenomenon in human myocytes^11^, and the molecular constituents of all three major depolarizing sarcolemmal currents, I_CaL_, I_Na_, and I_NaCa_, are thought to be relatively consistent across species^1^. To say that conserved sequences implied conservation of overall structure or indeed ion channel function in all cases would be an oversimplification, as differences have been confirmed e.g.^41,42^. However, the relative conservation of expressed ion channel proteins across mammalian species provides a solid basis for application of our methodology for translation of drug effects between species, given an understanding of the divergence, as well as sufficient data for parameterization and validation.

## Methods

Mathematical models of the membrane potential of excitable cells are written on the form

1 $$\frac{dv}{dt}=-\,\sum _{x}\,{I}_{x},$$

see e.g.^43–46^. Here, *v* is the membrane potential (in mV), *t* denotes time (in ms) and *I*_*x*_ are the membrane currents (in A/F). Each individual current can be written on the form

2 $${I}_{x}=\frac{{N}_{x}}{A{C}_{m}}{g}_{0}^{x}{o}_{x}(v-{E}_{x}),$$

where *A* is the area of the cell membrane (in *μ*m^2^) and *C*_*m*_ is the specific capacitance of the cell membrane (in pF/*μ*m^2^); both *A* and *C*_*m*_ are common parameters for every currents in a given type of cardiomyocyte. Furthermore, *N*_*x*_ is the number of channels of type *x*, ${g}_{0}^{x}$ is the conductance of a single open channel (in nS), *o*_*x*_ is the unitless open probability of the channel. Finally, *E*_*x*_ is the electrochemical equilibrium potential of the channel (in mV). By introducing the constant

3 $${\rho }_{x}=\frac{{N}_{x}}{A{C}_{m}},$$

we get the simpler expression

4 $${I}_{x}={\rho }_{x}{g}_{0}^{x}{o}_{x}(v-{E}_{x}).$$

**Figure 2:**
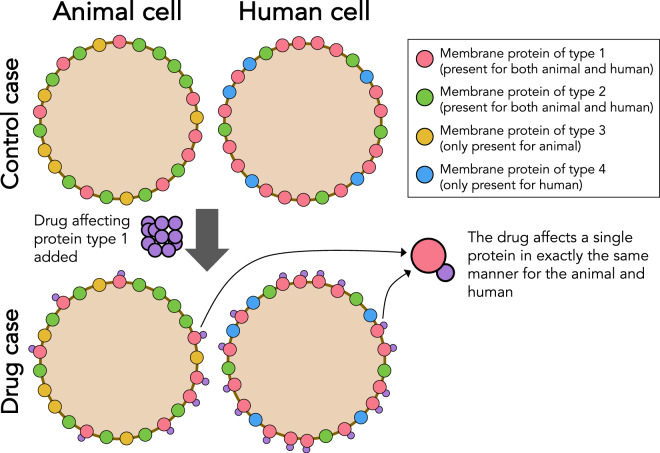
Upper left; illustration of an animal cardiomyocyte that express three types of ion-channels (proteins) in the myocyte membrane surface (sarcolemma). Two of the proteins are also present in the membrane of the human cardiomyocyte (upper right). Note that the human ventricular myocyte also express an additional type of protein *not present* in the animal cell. In the lower panels, both the animal and the human cardiomyocytes are subjected to a blocking drug that binds to one of the protein types. An essential assumption for allowing quantitative translation of animal measurements to estimates of the effect of human cells is that, at the level of a single protein, the effect of the drug is independent of whether the protein is expressed in human or an animal cell membrane.

In (4) it is important to note that the characteristics of the dynamics of the specific ion channel under consideration is given by the open probability *o*_*x*_; the rest of the expression is either constants or the common term (*v* − *E*_*x*_). The dynamics of *o*_*x*_ can be modelled using a Markov scheme (see e.g.^43,44,47^). Here, it is critical to acknowledge that if the ion channel in two different species are identical, then so is the Markov model governing their dynamics. The diagram in Fig. 2 illustrates the assumptions underlying the translation from animal to human.

The effect of a specific channel blocker is often expressed in terms of an IC_50_ value (see e.g.^48^; here we use the Hill coefficient 1). The IC_50_ value indicates the concentration that reduces the channel conductance by 50%. We assume that when a channel blocker is applied, the current takes the form

5 $${I}_{x}(D)={\rho }_{x}{g}_{x}^{0}(D){o}_{x}(v-{E}_{x}),$$

where we have defined the conductance of the single channel to be

6 $${g}_{x}^{0}(D)=\frac{{g}_{0}^{x}}{1+{\varepsilon }_{x}D}.$$

In (6), we have introduced the parameter *ε*_*x*_ = 1/IC_50_; here *D* is the drug concentration given in the same unit as the IC_50_ (often *μ*M). Clearly, ${I}_{x}({{\rm{IC}}}_{50})=\frac{1}{2}{I}_{x}(0),$ where *I*_*x*_(0) is the current in baseline or control case. Note from (5) that we assume that the blocker only affects the maximum conductance of the single channel. This is a known oversimplification since the effects of drugs may be much more complex; see e.g.^47,49,50^. However, this assumption greatly simplifies the identification of the drug effect from measurements, and it is sufficient for our purposes.

### IC50 for animal current = IC50 for human current

When a drug effect on an ion channel in an animal myocyte is detected, the key observation for translation to humans is that, given the assumption of functional invariance of channels between species (see Fig. 2), the effect of the drug on a single channel protein must be the same whether the protein is expressed in an animal or human. More specifically, if a current in an animal cardiomyocyte is given by

7 $${I}_{A}={\rho }_{A}{g}_{0}o(v-E),$$

then the associated human current is given by

8 $${I}_{H}={\rho }_{H}{g}_{0}o(v-E).$$

If a drug is added to the animal channel with a given IC_50_ value, then, as above, *g*_0_ of (7) is replaced by

9 $${g}^{0}(D)=\frac{{g}_{0}}{1+\varepsilon D},$$

where *ε* = 1/IC_50_. But since the conductance of the single channel is the same for the animal current and the human current, the single channel conductance of the human model (8) is also replaced by the expression given in (9). Hence, when a drug of concentration *D* is added we have the currents

10 $${I}_{A}(D)={\rho }_{A}\frac{{g}_{0}}{1+\varepsilon D}o(v-E),$$

11 $${I}_{H}(D)={\rho }_{H}\frac{{g}_{0}}{1+\varepsilon D}o(v-E).$$

Therefore, ${I}_{A}({{\rm{IC}}}_{50})=\frac{1}{2}{I}_{A}(0)$, and, similarly, ${I}_{H}({{\rm{IC}}}_{50})=\frac{1}{2}{I}_{H}(0)$, and thus the IC_50_ value of the animal and human current is the same. In principle, this key observation also is valid when the drug effect is modeled as a part of a complex Markov model, since the effect of the drug, on the single channel, again would be the same for animals and humans as long as the single ion channels are identical.

Details regarding the specific choice of action potential model used in our computations and the method used to identify drug effects on single ion channels from membrane potential measurements are given in the Supplementary information.

## Supplementary Information

### Translating from animal to human

Let us consider a very simple situation where we assume that both the animal myocyte action potential (a) and the human action potential (h) are generated by only two currents. The mathematical models takes then the form

12 $$\frac{d{v}^{y}}{dt}=-{\rho }_{1}^{y}\frac{{g}_{0}^{1}}{1+{\varepsilon }_{1}D}{o}_{1}^{y}({v}^{y}-{E}_{1})-{\rho }_{2}^{y}\frac{{g}_{0}^{2}}{1+{\varepsilon }_{2}D}{o}_{2}^{y}({v}^{y}-{E}_{2}),$$

where *y* is either *a* for animal or *h* for human. Here, clearly ${\rho }_{1}^{y}$, ${\rho }_{2}^{y}$ are different for *y* = *a* and *y* = *h* due to differences in the ion channel density. However, since the ion channels are the same, the Markov model governing the ${o}_{1}^{y}$, for *y* = *a* and *y* = *h* are the same.

Suppose now that we have measured an animal dataset sufficient to determine the coefficients *ε*_1_ and *ε*_2_. We immediately observe that we have a complete mathematical model for both the animal and the human action potentials. Using these principles, we can compute the biomarkers of interest for the human case.

Certainly, mathematical models used to represent action potentials are much more complex than the template model given by (12) in the main text; see e.g.^11,51,52^. In our computations, we use the Base model developed in^13^, as it systematically uses the same model for the identical ion channel in two different species. Note that in^13^ the results of the Base model is compared to the results of the Paci model (for immature cells) and to the Grandi *et al*. model and the ORd model (adult human cells).

### Example

In order to explain and illustrate how it is possible to use animal data to gain quantitative insight concerning effects on human ventricular myocytes, we will present a very simple example. Let us for the sake of argument assume that an animal myocyte action potential is governed by

$$\frac{dv}{dt}=-\,{g}_{1}{{\rm{I}}}_{{\rm{Na}}}-{g}_{2}{{\rm{I}}}_{{\rm{CaL}}}-{g}_{3}{{\rm{I}}}_{{\rm{Ks}}}$$

and the analogous human AP is governed by the very simple model,

$$\frac{dv}{dt}=-\,{k}_{1}{{\rm{I}}}_{{\rm{Na}}}-{k}_{2}{{\rm{I}}}_{{\rm{CaL}}}-{k}_{3}{{\rm{I}}}_{{\rm{Kr}}}.$$

Here *g*_1_, *g*_2_, *g*_3_, *k*_1_, *k*_2_, *k*_3_ are conductances and we assume that they are of significant magnitude. Suppose, for instance, that a drug is applied to the animal ventricular myocytes and inversion of the measured data suggests that the drug is a calcium blocker with the IC_50_ value given by 1/*ε*_CaL_. Then the animal model in the setting when the drug has been applied is given by

13 $$\frac{dv}{dt}=-\,{g}_{1}{{\rm{I}}}_{{\rm{Na}}}-\frac{{g}_{2}}{1+D{\varepsilon }_{{\rm{CaL}}}}{{\rm{I}}}_{{\rm{CaL}}}-{g}_{3}{{\rm{I}}}_{{\rm{Ks}}},$$

where *D* denotes the concentration of the applied drug. Since the IC_50_ value of the single channel is the same for the animal and the human myocytes, the human model takes the form

$$\frac{dv}{dt}=-\,{k}_{1}{{\rm{I}}}_{{\rm{Na}}}-\frac{{k}_{2}}{1+D{\varepsilon }_{{\rm{CaL}}}}{{\rm{I}}}_{{\rm{CaL}}}-{k}_{3}{{\rm{I}}}_{{\rm{Kr}}}.$$

This model can be used to compute chosen biomarkers of interest for the human action potential. In this particularly simple situation, we can conclude that:

1. One can use the animal datasets to reveal drug effect on human I_Na_ and I_CaL_.
2. One cannot use the animal to get any information about human I_Kr_ because the animal does not have I_Kr_.
3. The I_Ks_ current in the animal does not create difficulties for the measurements.

### The base model of the action potential

In all of our simulations, we use the Base model derived in^13^. The parameters used to model human, dog, zebrafish, rabbit and guinea pig action potentials are given in Table 4. The remaining parameter values are specified in^13^, except that the intracellular potassium concentration is increased from 120 mM to 145 mM for the dog case in order to match the resting potential observed in the data from^7^. Note also that because zebrafish cardiomyocytes have been shown to exhibit a robust T-type calcium current (*I*_CaT_)^17^, the Base model from^13^, when utilized for the zebrafish simulations, is extended to include a T-type calcium current from^53^ of the form

$${I}_{{\rm{CaT}}}={g}_{{\rm{CaT}}}\cdot d\cdot f\cdot (v-{E}_{{\rm{Ca}}}),$$

where *g*_CaT_ is the conductance of the channels and *E*_Ca_ is the Nernst equilibrium potential for calcium (see^13^). Here, *d* and *f* are gating variables governed by

$$\frac{dd}{dt}=\frac{{d}_{\infty }-d}{{\tau }_{d}},\,{d}_{\infty }=\frac{1}{1+{e}^{-(v+26.3)/6}},$$

$${\tau }_{d}=\frac{1}{1.068{e}^{(v+26.3)/30}+1.068{e}^{-(v+26.3)/30}},$$

$$\frac{df}{dt}=\frac{{f}_{\infty }-f}{{\tau }_{f}},\,{f}_{\infty }=\frac{1}{1+{e}^{(v+61.7)/5.6}},$$

$${\tau }_{f}=\frac{1}{0.0153{e}^{-(v+61.7)/83.3}+0.015{e}^{(v+61.7)/15.38}}.$$

**Table 4:** Parameter values of the Base model used to represent different species.

Parameter	Human	Dog	Zebrafish	Rabbit	Guinea pig
*g*_Kr_ (mS/*μ*F)	0.033	0.016	0.044	0.087	0.06
*g*_CaL_ (nL/(*μ*F ms))	0.17	0.077	0.086	0.25	0.21
*g*_Na_ (mS/*μ*F)	5	1.4	2.2	2.3	0.90
*g*_Ks_ (mS/*μ*F)	0.003	0.1	0.0024	0.016	0.69
*g*_KL_ (mS/*μ*F)	0.074	0.52	0.86	0.35	0.85
*g*_to_ (mS/*μ*F)	0.54	0.31	0.0035	0.17	0.00
*g*_NaL_ (mS/*μ*F)	0.025	0.0042	0.028	0.027	0.03
${\bar{I}}_{{\rm{NaCa}}}$ (*μ*A/*μ*F)	4.9	5.9	4.2	9.8	15.86
${\bar{I}}_{{\rm{NaK}}}$ (*μ*A/*μ*F)	1.8	1.4	9.2	2.8	1.62
*g*_bCl_ (mS/*μ*F)	0.0056	0.00013	0.00087	0.011	0.00
*g*_bCa_ (mS/*μ*F)	0.00055	0.00065	0.0019	0.0005	0.00
${\bar{I}}_{{\rm{pCa}}}$ (*μ*A/*μ*F)	0.068	0.58	0.056	0.064	0.60
*g*_CaT_ (mS/*μ*F)	0	0	0.056	0	0

The remaining parameter values used in the simulations are as specified in^13^.

### Inversion of data

The inversion of data from dog, zebrafish and rabbit action potentials is performed using the inversion procedure described in^13^. In the inversion, the adjustment factors *λ*_Kr_, *λ*_CaL_, *λ*_Na_, *λ*_Ks_, *λ*_Kl_, *λ*_to_, *λ*_NaL_, *λ*_NaCa_, *λ*_NaK_, *λ*_bCl_, *λ*_bCa_, and *λ*_pCa_ for the currents *I*_Kr_, *I*_CaL_, *I*_Na_, *I*_Ks_, *I*_Kl_, *I*_to_, *I*_NaL_, *I*_NaCa_, *I*_NaK_, *I*_bCl_, *I*_bCa_, and *I*_pCa_, are treated as free parameters, in the zebrafish case, the adjustment factor λCaT for the current ICaT is also treated as a free parameter in the inversion, in addition to *ε*_Kr_ (and *ε*_CaL_ for sotalol), representing the effect of the drug (see (6)).

#### Cost function definition

In the inversion, we minimize a cost function of the form

14 $$H(\lambda ,\varepsilon )=\sum _{d}\,\sum _{j}\,{w}_{d,j}{({H}_{j}(\lambda ,\varepsilon ,{D}_{d}))}^{2},$$

where *d* numbers the drug doses, *D*_*d*_, included in the data set (including the control case), *j* numbers the different cost function terms, *H*_*j*_, included in the cost function, and *w*_*d*,*j*_ are specified weights for each of the cost function terms and each of the doses. The cost function includes the terms

$${H}_{{\rm{APD}}p}(\lambda ,\varepsilon ,{D}_{d})=\frac{|{\rm{APD}}p(\lambda ,\varepsilon ,{D}_{d})-{\rm{APD}}{p}^{\ast }({D}_{d})|}{|{\rm{APD}}{p}^{\ast }({D}_{d})|},\,{\rm{for}}\,p=20,30,\ldots ,90,$$

$${H}_{{v}_{{\rm{\max }}}}(\lambda ,\varepsilon ,{D}_{d})=\frac{|{v}_{{\rm{\max }}}(\lambda ,\varepsilon ,{D}_{d})-{v}_{{\rm{\max }}}^{\ast }({D}_{d})|}{|{v}_{{\rm{\max }}}^{\ast }({D}_{d})|},$$

$${H}_{{v}_{{\rm{\min }}}}(\lambda ,\varepsilon ,{D}_{d})=\frac{|{v}_{{\rm{\min }}}(\lambda ,\varepsilon ,{D}_{d})-{v}_{{\rm{\min }}}^{\ast }({D}_{d})|}{|{v}_{{\rm{\min }}}^{\ast }({D}_{d})|},$$

where APD *p* is the action potential duration at *p* percent repolarization (see^13^). Furthermore, *v*_min_ and *v*_max_ are the minimum and maximum values of the membrane potential, respectively. The terms marked by an * represent values computed from measured data, and the remaining terms are computed from simulations of the model defined by *λ* and *ε*.

#### Cost function weight

We use the weight 2 for ${H}_{{v}_{{\rm{\max }}}}$, ${H}_{{v}_{{\rm{\min }}}}$ and *H*_APD50_, the weight 5 for *H*_APD90_ and the weight 1 for the remaining terms. In addition, the weights for the control case, *w*_0,*j*_, are multiplied by the total number of doses included in the data set (including the control case).

#### Minimization procedure

In order to minimize the cost function (14), we apply the continuation-based minimization procedure from^13^ using 10 iterations with 100 randomly chosen initial guesses. The initial guesses for *λ* are chosen within 10% above or below the optimal values from the previous iteration, and the initial guesses for *ε*_*m*_ are chosen within [*ε*_*m*−1_/5, 5*ε*_*m*−1_], where *ε*_*m*−1_ is the optimal *ε* from the previous iteration. From these initial guesses we run 15 iterations of the Nelder-Mead algorithm^54^. The starting points for the first iteration of the continuation method are adjusted by hand.

### Application of the method to generated data

In the main text, we have shown that we are able to use animal datasets (from dog, zebrafish and rabbit) to estimate the effect of *I*_Kr_ blockers on healthy human ventricular myocytes. However, available published data is limited and we therefore want to indicate that the methodology works more generally than we have been able to prove using available datasets. In Table 5, we have additionally used simulations to generate data. More specifically, we use the zebrafish model to estimate the effect for the human model of blocking the sodium current and the calcium current by 50%. In these inversions, *λ*_Na_, *λ*_CaL_ and *λ*_Kr_ and *ε*_Na_, *ε*_CaL_ and *ε*_Kr_ are treated as free parameters. In the generation of the zebrafish data, *I*_Na_, *I*_CaL_ and *I*_Kr_ are all increased by 20%. Furthermore, in order to detect changes in the sodium current, we extend the cost function to include the term

$${H}_{{\rm{dvdt}}}(\lambda ,\varepsilon ,{D}_{d})=\frac{|\frac{dv(\lambda ,\varepsilon ,{D}_{d})}{dt}-\frac{d{v}^{\ast }({D}_{d})}{dt}|}{|\frac{d{v}^{\ast }({D}_{d})}{dt}|},$$

measuring differences in the upstroke velocity. The results in Table 5 illustrate that the method presented here has wide applicability, provided that the ion currents under consideration have significant impact on both animal and human action potentials.

**Table 5:** Effect of 50% block of *I*_Na_ and *I*_CaL_ on APD50 and the maximal upstroke velocity of the action potential in zebrafish and human models.

	Zebrafish control “measured”	Zebrafish drug “measured”	Human control “measured”	Human drug “measured”	Human control model	Human drug, estimated based on zebrafish data
APD50 (ms)	110	−7.9%	214	−10.5%	214	−9.3%
${\left(\frac{dv}{dt}\right)}_{{\rm{\max }}}$ (mV/ms)	102	−49.1%	195	−43.6%	195	−42.7%

The zebrafish and human data is generated from simulations of the zebrafish and human models specified in Section 5.2. In addition, the effect of the drug is estimated based on the zebrafish case and mapped to the human case in the rightmost column.

### Quantitative translation from guinea pig to zebrafish

The purpose of this paper is to show how a computational approach can be used to translate measured drug responses for animals to corresponding drug responses for humans. We have demonstrated how data from dogs, zebrafish and rabbits can be translated into predictions of human drug responses. In Table 6, we demonstrate how the same approach may be used to translate the effect of a drug from one animal to another. In this case, we consider data from^18^ of guinea pig cardiomyocytes exposed to four different doses of the *I*_Kr_ blocker E-4031. In addition, measured zebrafish data of 1 *μ*M E-4031 are provided in^17^. Based on the measured guinea pig data, the mathematical method estimates an IC_50_ value of 27 nM for block of *I*_Kr_ by E-4031. This is in good agreement with values found in literature (10–397 nM^25–30^) as well as with the IC_50_ value of 44 nM identified based on zebrafish data in the main text. In Table 6, we observe that the method predicts a 41.7% increase in APD50 and a 35.2% increase in APD90 for zebrafish cardiomyocytes exposed to 1 *μ*M E-4031, in line with the measured increases of 38.7% and 35.4% for APD50 and APD90, respectively.

**Table 6:** APD values computed from measured and simulated action potentials in the control case and in the presence of the *I*_Kr_ blocker E-4031.

	Guinea pig control measured	Guinea pig drug measured	Zebrafish control measured	Zebrafish drug measured	Zebrafish control model	Zebrafish drug estimated based on guinea pig data
APD50 (ms)	156	+10.2% (1 nM)	108		111	+1.0% (1 nM)
		+16.4% (30 nM)				+17.8% (30 nM)
		+30.1% (100 nM)				+30.4% (100 nM)
		+37.2% (1 *μ*M)		+38.7% (1 *μ*M)		+41.7% (1 *μ*M)
APD90 (ms)	178	+9.9% (1 nM)	133		133	+0.8% (1 nM)
		+16.6% (30 nM)				+15.0% (30 nM)
		+31.5% (100 nM)				+25.6% (100 nM)
		+38.4% (1 *μ*M)		+35.4% (1 *μ*M)		+35.2% (1 *μ*M)

The guinea pig data are from^18^ and include the control case and the four drug doses 1 nM, 30 nM, 100 nM and 1 *μ*M. The zebrafish data are from^17^ and include the control case and the case of 1 *μ*M E-4031^55^.
